# Li-Fraumeni-like syndrome associated with a large *BRCA1* intragenic deletion

**DOI:** 10.1186/1471-2407-12-237

**Published:** 2012-06-12

**Authors:** Amanda Gonçalves Silva, Ingrid Petroni Ewald, Marina Sapienza, Manuela Pinheiro, Ana Peixoto, Amanda França de Nóbrega, Dirce M Carraro, Manuel R Teixeira, Patricia Ashton-Prolla, Maria Isabel W Achatz, Carla Rosenberg, Ana C V Krepischi

**Affiliations:** 1 International Center for Research and Training, A. C. Camargo Cancer Hospital, São Paulo, Brazil; 2 National Institute of Science and Technology in Oncogenomics, São Paulo, Brazil; 3 Experimental Research Center Hospital de Clínicas de Porto Alegre, Rio Grande do Sul, Brazil; 4 Post-Graduate Course in Medicine, Medical Sciences, Federal University of Rio Grande do Sul, Porto Alegre, Brazil; 5 Department of Genetics and Evolutionary Biology, Institute of Biosciences, University of São Paulo, São Paulo, Brazil; 6 Department of Genetics, Portuguese Oncology Institute, Porto, Portugal; 7 Biomedical Sciences Institute (ICBAS), University of Porto, Porto, Portugal; 8 Department of Genetics and Post-Graduate Program in Genetics and Molecular Biology, Federal University of Rio Grande do Sul and Medical Genetics Service, Hospital de Clínicas de Porto Alegre, Porto Alegre, Brazil; 9 Hospital A. C. Camargo, Rua Taguá, 440 – Liberdade – CEP 01508-010, São Paulo, Brazil

**Keywords:** Breast cancer, Copy number variation, MLPA, *BRCA1* microdeletion, Li-Fraumeni syndrome

## Abstract

**Background:**

Li-Fraumeni (LFS) and Li-Fraumeni-like (LFL) syndromes are associated to germline *TP53* mutations, and are characterized by the development of central nervous system tumors, sarcomas, adrenocortical carcinomas, and other early-onset tumors. Due to the high frequency of breast cancer in LFS/LFL families, these syndromes clinically overlap with hereditary breast cancer (HBC). Germline point mutations in *BRCA1*, *BRCA2*, and *TP53* genes are associated with high risk of breast cancer. Large rearrangements involving these genes are also implicated in the HBC phenotype.

**Methods:**

We have screened DNA copy number changes by MLPA on *BRCA1*, *BRCA2*, and *TP53* genes in 23 breast cancer patients with a clinical diagnosis consistent with LFS/LFL; most of these families also met the clinical criteria for other HBC syndromes.

**Results:**

We found no DNA copy number alterations in the *BRCA2* and *TP53* genes, but we detected in one patient a 36.4 Kb *BRCA1* microdeletion, confirmed and further mapped by array-CGH, encompassing exons 9–19. Breakpoints sequencing analysis suggests that this rearrangement was mediated by flanking *Alu* sequences.

**Conclusion:**

This is the first description of a germline intragenic *BRCA1* deletion in a breast cancer patient with a family history consistent with both LFL and HBC syndromes. Our results show that large rearrangements in these known cancer predisposition genes occur, but are not a frequent cause of cancer susceptibility.

## Background

Germline mutations of the tumor suppressor gene *TP53* account for more than half of the families with classic Li-Fraumeni syndrome (LFS) [1], which is an inherited condition characterized by the development of sarcomas and other early-onset tumors, including breast cancer [2,3]. Families presenting incomplete features of LFS are referred as having Li-Fraumeni-like syndrome (LFL). Depending on the criteria adopted to classify the cancer phenotype in a given family, up to 22% of LFL pedigrees have detectable *TP53* mutations [4-6]. Several cancer predisposition syndromes that involve breast cancer have been described to date, and include, in addition to LFS/LFL, the hereditary breast and ovarian cancer (HBOC), hereditary diffuse gastric cancer, and the Cowden and Peutz-Jeghers syndromes [7]. Due to the high frequency of breast and other cancers in LFS/LFL individuals, there may be an overlap of phenotypes, and often some families fulfill genetic testing criteria for more than one hereditary breast cancer syndrome [1,8,9].

Several studies have investigated the frequency of *BRCA1/BRCA2* and *TP53* germline mutations in families with multiple early-onset breast cancers [6,8,10,11]. Approximately 5-10% of breast cancer is estimated to result from dominant mutations in known single genes [12-14], particularly in the *BRCA1* or *BRCA2* genes. Germline *TP53* mutations have been considered to be responsible for only a small fraction of the hereditary breast cancer cases overall [15], and have mostly been described in families with the other core-cancers of LFS/LFL [1,8,9]. Germline mutations of the *BRCA2* gene have been described in families presenting both breast cancer and sarcomas, suggesting that *BRCA2* mutations account for a proportion of LFS/LFL families negative for *TP53* mutations [16,17]. As far as we are aware, germline *BRCA1* mutations have not been detected in LFS/LFL kindreds, not even among families presenting a complex cancer history consistent both with LFL and other syndromes that constitute the HBC phenotype [6,8,11,18].

All known breast cancer susceptibility genes present germline point mutations in only approximately 20-25% of the cases fulfilling the criteria for genetic testing [12]. Gene rearrangements can contribute to disease through different mechanisms, resulting in either imbalance of gene dosage or gene disruption, and they are not usually detected by routine molecular diagnostic methods such as gene sequencing. In particular, large rearrangements, most often deletions, have been reported as a cause of cancer susceptibility, occurring in at least 30% of highly penetrant Mendelian cancer-predisposing genes [19].

*BRCA1* germline rearrangements have been implicated in up to 30% of HBC families in certain populations [19-23]. The aim of the present study was to determine the frequency of germline copy number changes of *TP53BRCA1*, and *BRCA2* genes in breast cancer patients with clinical diagnosis of Li-Fraumeni or Li-Fraumeni-like syndrome, and without detectable germline *TP53* point mutations.

## Results

All studied patients were females affected by breast cancer, two of them with bilateral disease, and 11 (45.8%) with more than one primary tumor. The average age at breast cancer diagnosis was 41 years (SD: 11.5; range: 26–61 years). Nineteen of the 23 families met genetic testing criteria for both LFL and another hereditary breast cancer syndrome (Table 1); two families met criteria for both classic LFS and another hereditary breast cancer syndrome, and two fulfill only the criteria for LFL.

**Table 1:** Characteristics of the probands: clinical phenotype, type of tumor and age of diagnosis (years)

**Individual Code**	**Classification**	**Breast tumor (age at diagnosis)**	**Other tumors (age at diagnosis)**
**Y6T000**	Birch	breast (79)	Lymphoma (73), skin (81)
**Y29T000**	Birch/HBC	breast (26)	Osteosarcoma (19), soft tissue sarcoma (23), head/neck (24)
**Y36T000**	Birch/HBC	breast (44)	
**Y41T000**	Chompret/HBC	breast (28)	Osteosarcoma (8)
**Y51T000**	Eeles1	breast (53)	
**Y54T000**	Eeles1/HBC	breast (41)	Endometrium (44)
**Y83T000**	Chompret/HBC	breast (45)	Soft tissue sarcoma (21)
**Y93T000**	LFS/HBC	breast (42)	
**Y95T000**	Eeles2/HBC	breast (36)	
**Y101T000**	Eeles1/HBC	breast (48)	Thyroid (52)
**Y110T000**	Eeles1/HBC	breast (36)	
**Y112T000**	Chompret/HBOC	breast (34)	
**Y115T000**	Chompret/HBC	breast (36)	
**Y116T000**	Eeles2/HBC	breast (48)	
**Y117T000**	Eeles1/HBC	breast (44)	
**Y122T000**	Eeles1/HBC	breast (61)	Colorectal cancer(68)
**Y123T000**	Eeles1/HBC	breast bilateral (37)	
**Y126T000**	Chompret/HBC	breast (39)	Lymphoma (23), skin (40)
**Y135T000**	Eeles1/HBC	breast (30)	
**Y143T000**	Eeles1/HBC	breast (42)	
**Y145T000**	Chompret/HBC	breast, bilateral (36; 36)	
**Y147T000**	Chompret/HBC	breast (35)	Melanoma (36)
**Y152T000**	LFS/HBC	breast (38)	Skin (36)

**Figure 1:**
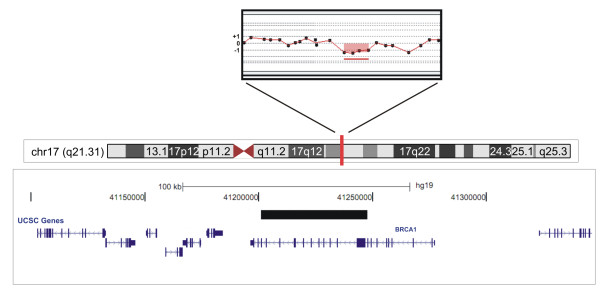
**Mapping of the intragenic*BRCA1*deletion detected in a patient with multiple primary tumors and a cancer family history fulfilling criteria for*TP53*and*BRCA*testing.** In the upper panel, the array-CGH profile of a region at chromosome band 17q21.31, showing a heterozygous loss in copy number (red bar) of a genomic segment (image adapted from the Genomic Workbench software, Agilent Technologies). The lower panel displays the deleted segment (solid black bar) in the context of the genomic region, encompassing exons 9–19 of the *BRCA1* gene according to the analysis of breakpoint sequencing data (image adapted from UCSC Genome Bioinformatics, http://genome.ucsc.edu, Build 37.1).

In the MLPA analysis none of the patients showed *TP53, or BRCA2* deletions or duplications. We identified a single patient carrying a heterozygous intragenic *BRCA1* microdeletion (Y54). Analysis using two different sets of MLPA probes (kits P087 and P002) and array-CGH allowed confirming a deletion that spanned from exon 9 to 19 (Figure 1 depicts the chromosome 17q21.31 array-CGH profile of the patient, indicating the position of the *BRCA1* microdeletion). We tested two non-affected relatives of patient Y54 (III.13 and III.16) and found that one of them carries the *BRCA1* deletion (III.16). Unfortunately, affected relatives of the patient Y54 could not be investigated for the presence of the *BRCA1* deletion either because they were deceased or were not available.

**Figure 2:**
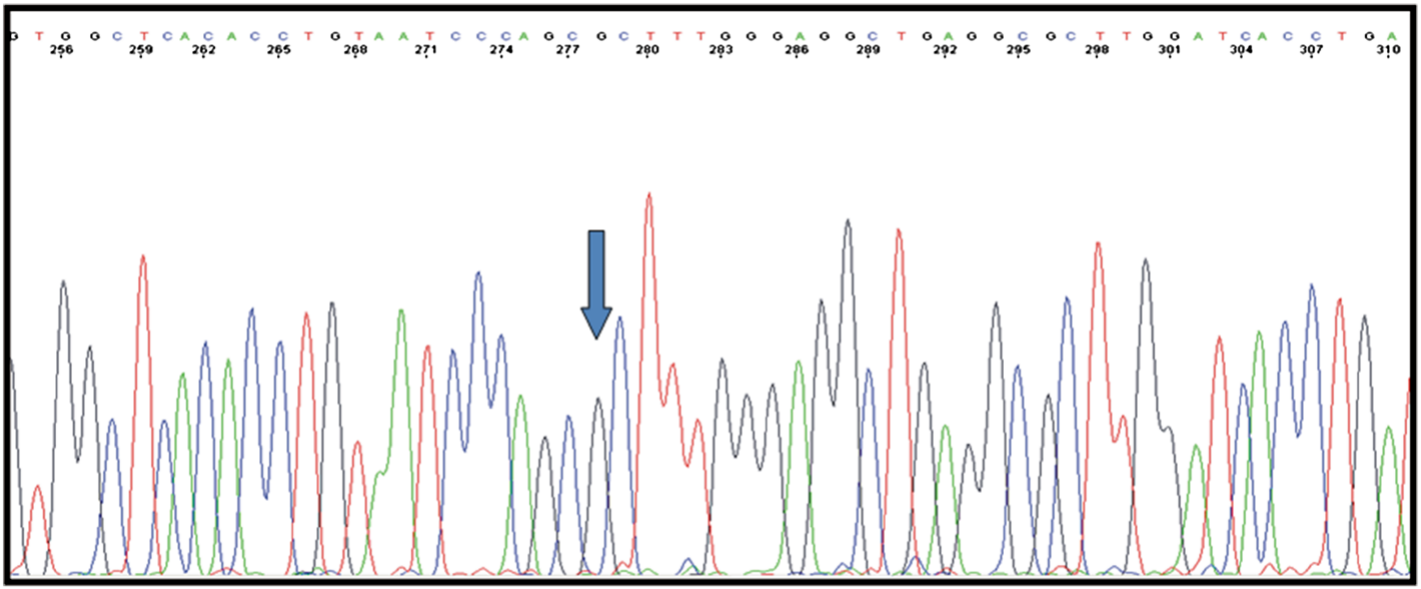
**Breakpoint sequencing analysis.** Eletropherogram showing the g.29197_65577del36381 mutation in the *BRCA1* sequence; the intron 8 sequence is followed by intron 19 sequence. The blue arrow represents the inferred breakpoint.

The DNA fragment containing the rearrangement breakpoints was sequenced and the results showed that the deletion starts at intron 8 and ends at intron 19 of the *BRCA1* gene, resulting in a deletion-block identified as: g.29197_65577del36381 (Figure 2). Detailed *in silico* assessment of the genomic sequences surrounding the breakpoints showed that consensus *Alu* sequences flanked them.

**Figure 3:**
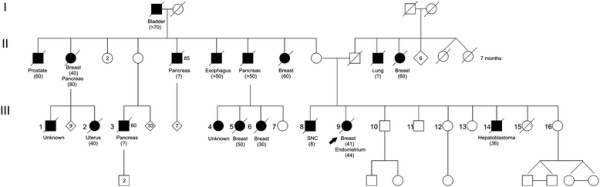
**Pedigree of the family with a large*BRCA1*rearrangement.** Type of cancer is indicated under the subjects and the age of diagnosis is shown in brackets.

Clinically, this family fulfilled genetic testing criteria for both hereditary breast and ovarian cancer (HBOC) and LFL (Eeles 1 criteria) syndromes; the cancer family history was significant for the presence of two individuals with multiple primary tumors, including the proband (Figure 3).

## Discussion

In families with a breast cancer history that suggests the involvement of high risk genes such as *TP53*, *BRCA1* and *BRCA2*, a more extensive analysis of these genes should be considered. In this study we have screened three major breast cancer predisposition genes for copy number changes in a group of 23 breast cancer patients with the clinical diagnosis of LFS/LFL who had no germline *TP53* point mutations.

We did not identify large rearrangements encompassing *TP53*, which is in line with previous reports of low prevalence of such alterations, encountered in less than 5% of LFS/LFL families [24,25].

Similarly, large rearrangements in other breast cancer predisposition genes seem to be infrequent. A few *BRCA2* deletions have been previously reported in families with male breast cancer [26], and contribute to inactivate this gene in breast cancer families [21,27]. Rearrangements affecting the *BRCA2* gene have also been reported in breast/sarcoma families, causing a Li–Fraumeni type of cancer pattern [16]. Although none of the families included in this study had male breast cancer cases, nine of them had a breast cancer/sarcoma phenotype; however, no *BRCA2* rearrangements were identified, which may be related to the relatively small sample size.

*BRCA1* rearrangements, on the other hand, are more prevalent mostly due to the high density of *Alu* elements throughout the *BRCA1* locus [28]*.* A large study by Walsh *et al* (2006) [11] suggested that the mutation spectra of *BRCA1/BRCA2* includes several genomic rearrangements, and those alterations seem to be particularly frequent in certain populations (due to founder effect), and in families presenting individuals with multiple primary tumors [20,21,29,30]. Indeed, the “multiple primary tumors” phenotype was observed in the *BRCA1* rearrangement-positive family identified in our series. Interestingly, the *BRCA1* microdeletion identified here appears to be the same as the one identified in a breast cancer Italian patient [20]. Our patient is originally from southern Brazil, and since Italians have strongly contributed to the ethnic make-up of southern Brazilian population [31] it is possible that the Brazilian and the Italian patients have a common ancestry. Considering that we could not establish the parental origin of the rearrangement, this large genomic deletion may represent a breast cancer susceptibility allele rather than a more general cancer predisposition factor.

This study contributes to the understanding of the etiology of cancer susceptibility in Li-Fraumeni (LFS) and Li-Fraumeni-like (LFL) families, and their possible relation to large genomic rearrangements in high risk breast cancer susceptibility genes.

## Conclusion

In patients with a cancer family history consistent with genetic testing criteria for multiple breast cancer syndromes, a comprehensive investigation, including full gene sequencing and rearrangement screening of multiple *loci* may be necessary to determine the precise molecular mechanisms underlying the disease. However, as illustrated with this study, in many families with cancer histories clearly indicative of hereditary cancer predisposition, the disease-causing molecular mechanisms remain elusive. Thus, despite the availability of extensive genotyping and sequencing approaches, determination of the precise pathogenic mechanisms of hereditary cancer in many cases is still a significant challenge.

## Methods

### Patients

The research protocol was approved by the institutional ethics committees of the participating Institutions (Protocol numbers 1175/08 and GPPG-HCPA 04–081), and recruitment of patients was done after signature of informed consent. DNA samples from 23 patients were obtained from peripheral blood; sample quality was assessed using Nanodrop and molecular weight was checked by electrophoresis in 0.8% agarose gels. *TP53* mutation testing was previously performed by direct sequencing of exons 2–11, using the protocols published in http://www-p53.iarc.fr/p53sequencing.html[24].

Family history was recorded in detailed pedigrees with information traced as far backwards and laterally as possible, extending to paternal lines and including a minimum of three generations. Confirmation of the family history of cancer was attempted in all cases and pathology reports, medical records and/or death certificates were obtained whenever possible.

We selected 23 breast cancer patients with an indication for *TP53* mutation testing due to a Li-Fraumeni or Li-Fraumeni-like phenotype according to the classical criteria [32] or at least one of the LFL definitions: Chompret, Birch or Eeles [4,33-35]. In all families, *TP53* mutation testing was negative [36]. Additionally, some of these families also fulfilled mutation testing criteria for other hereditary breast cancer syndromes, as described in the NCCN Practice Guidelines in Oncology – v.1.2010 [37].

Clinical features of the 23 probands are summarized in Table 1.

### Multiplex ligation probe amplification (MLPA)

Deletions and duplications affecting all coding exons of the *TP53* gene (12 probes) were investigated by MLPA [38](MRC-Holland, Amsterdam, The Netherlands, kit P056). MLPA experiments were performed in duplicates for each patient sample, with simultaneous analysis of DNA samples from two healthy individuals from the general population (negative controls), and two patients carrying previously characterized germline*TP53* rearrangements (positive controls: a Li-Fraumeni patient with an intragenic *TP53* deletion [39]; and a patient harboring a large 17p13 duplication from our in-house database). Deletions and duplications affecting *BRCA1* and *BRCA2* exons were also investigated by MLPA (MRC-Holland, Amsterdam, The Netherlands, kits P087 and P045, respectively; kit P002 was also used for confirmatory analysis of one detected *BRCA1* microdeletion); duplicated experiments were performed simultaneously in samples from patients, two healthy individuals, and samples previously identified as carrying large duplications encompassing the *BRCA1* and *BRCA2* genes (positive controls; patients from our in-house database).

The PCR-amplified fragments were separated by capillary electrophoresis on an ABI 3130 XL genetic analyzer (Applied Biosystems, Foster City, California), and analyzed using the Coffalyser software (MRC Holland). We performed direct normalization with control probes as normalization factor, using the median of all imported samples, and two standard deviations. Values >1.3 were considered as possible duplications, and deletions were considered for probes exhibiting values < 0.7. Using this analysis, alterations present in all positive controls were detected.

### Comparative genomic hybridization on microarrays (array-CGH)

Array-CGH analysis was performed as previously described [40] to confirm an intragenic *BRCA1* deletion detected by MLPA in one patient (Y54). We used a whole-genome 180 K platform (Agilent Technologies), according to the manufacturer’s instructions; a gain or loss in copy number was considered when the log_2_ ratio of the Cy3/Cy5 intensities of a given genomic segment was > 0.6 or < −0.8, respectively. As reference DNA, we used commercially available human Promega female DNA (Promega, Madison, WI, USA).

### Breakpoint Sequencing Analysis

To assess the microdeletion breakpoints, specific primers (forward: 5'- ACTCTGAGGACAAAGCAGCGGA -3'; reverse: 5'-GTGCCACCAAGCCCGGCTAA -3') were designed in order to amplify the breakpoint region of the *BRCA1* rearrangement (microdeletion involving the same exons described by [20]. A 450 bp fragment was detected only in the sample with the microdeletion, and absent in the normal controls. The 450 bp fragment was purified from the gel using the Gel Band Purification Kit (Illustra, GE Healthcare UK limited, Buckinghamshire, United Kingdom) and sequenced (forward and reverse) using the Big Dye V3.1 Terminator Kit (Applied Biosystems, Forster City, CA, USA) on an automated sequencer ABI Prism 310 Genetic Analyser (Applied BioSystems,) according to the manufacturer’s instructions.

We performed an *in silico* analysis of the genomic sequences surrounding the breakpoints using the RepeatMasker program ( http://www.repeatmasker.org/) that screens DNA sequences for interspersed repeats and low complexity DNA sequences.

## Abbreviations

LFS, Li-Fraumeni syndrome; LFL, Li-Fraumeni like syndrome; HBC, Hereditary breast cancer syndromes; HBOC, Hereditary breast and ovarian cancer syndrome; CNV, Copy number variation; Array-CGH, comparative genomic hybridization on microarrays; MLPA, Multiplex ligation probe amplification; NCCN, National Comprehensive Cancer Network; IARC, International Agency for Research on Cancer; UCSC, University of California, Santa Cruz.

## Competing interests

The authors declare that they have no competing interests.

## Authors’ contribution

AG carried out the molecular genetics studies and has drafted the manuscript. IPE carried out part of the MLPA assays and characterized the rearrangement breakpoints by sequencing. MS carried out part of the MLPA assays of the *TP53* gene. MP characterized the rearrangement breakpoints by sequencing. AP characterized the rearrangement breakpoints by sequencing. AFN participated of the clinical trial and classification of the families. DMC participated in the design of the study and revised the manuscript. MIWA and PAP were physicians responsible for the clinical trial, selection and classification of the families, and critically revised the manuscript. PAP and MT supervised the *BRCA1* MLPA and sequencing analyses and result interpretation. CR revised critically the manuscript. ACVK participated in the design of the study, performed part of the molecular genetics analysis, and helped to draft the manuscript. All authors read and approved the final manuscript.

### Links

National Comprehensive Cancer Network [ http://www.nccn.org/]

Database of Genomic Variants [ http://projects.tcag.ca/variation/]

International Agency for Research on Cancer [ http://www.iarc.fr/]

UCSC Genome Bioinformatics [ http://genome.ucsc.edu/]

## Pre-publication history

The pre-publication history for this paper can be accessed here:

http://www.biomedcentral.com/1471-2407/12/237/prepub
